# Computed Tomography-Derived Fractional Flow Reserve: Developing A Gold Standard for Coronary Artery Disease Diagnostics

**DOI:** 10.31083/j.rcm2510372

**Published:** 2024-10-22

**Authors:** Liangbo Hu, Yue Wang, Jingjing Rao, Lina Tan, Min He, Xiaocong Zeng

**Affiliations:** ^1^Department of Cardiology, The First Affiliated Hospital of Guangxi Medical University, 530021 Nanning, Guangxi, China; ^2^Guangxi Key Laboratory Base of Precision Medicine in Cardiocerebrovascular Diseases Control and Prevention & Guangxi Clinical, Research Center for Cardio-cerebrovascular Diseases, 530021 Nanning, Guangxi, China; ^3^School of Basic Medical Sciences, Guangxi Medical University, 530021 Nanning, Guangxi, China

**Keywords:** coronary CT angiography, coronary artery disease, fractional flow reserve, coronary computed tomography angiography

## Abstract

In recent years, a new technique called computed tomography-derived fractional flow reserve (CT-FFR) has been developed. CT-FFR overcomes many limitations in the current gold-standard fractional flow reserve (FFR) techniques while maintaining a better concordance with FFR. This technique integrates static coronary CT angiography data with hydrodynamic models, employing algorithms rather than guidewire interventions to compute the FFR. In addition to diagnosing coronary heart disease, CT-FFR has been applied in the preoperative risk assessment of major adverse cardiovascular events (MACEs) in organ transplantation and transcatheter aortic valve replacement (TAVR). Continuous advancements in CT-FFR techniques and algorithms are expanding their applicability to other methodologies. Subsequently, with robust clinical trial validation, CT-FFR can potentially supersede FFR as the primary “gatekeeper” for interventions.

## 1. Introduction

Obstructive coronary artery disease (CAD) significantly contributes to adverse cardiac outcomes in coronary heart disease (CHD). Historically, the invasive coronary angiography (ICA) criterion for assessing obstructive CAD was defined by a stenosis degree (DS) greater than 50%. Indeed, ICA remained the diagnostic gold standard until the development of computed tomography angiography (CTA) and fractional flow reserve (FFR) methodologies [1].

The FFR quantifies vascular blood flow ischemia, measuring the difference between the maximum blood flow in a diseased coronary artery and the maximum blood flow in the same vessel absent stenosis, with a theoretical ratio of 1.0. Since stenosis minimally affects intracoronary blood pressure, the FFR can be calculated as the arterial pressure ratio in the proximal coronary artery compared to that in the distal artery at the stenosis site. An FFR value of 0.8 indicates substantial impairment in myocardial function [2], which is critical for clinical diagnosis. As FFR is assessed during the congestion peak, it provides reliable readings and is minimally impacted by blood pressure and heart rate variations.

Angiographic findings and FFR flow pressure testing do not have a direct correlation. Vessels showing stenotic symptoms may not always correspond with ischemic FFR results. Stenoses between 50% and 90% often exhibit ischemic FFR values. However, FFR data are irrelevant in previous myocardial infarctions, stenosis exceeding ischemic FFR, or transient, reversible vascular damage or spasm. Traditionally, angiographic diagnosis was completed before percutaneous coronary intervention (PCI), with FFR testing conducted subsequently. In one trial, when the cutoff value was set at 0.80, significantly fewer stents were used in the FFR-guided group than in the angiography-guided group for PCI of non-infarct-related arteries (2.2 ± 1.1 vs. 2.5 ± 0.9, *p* <0.001) [3]. The DEFER trial [4] further validated the predictive value of the FFR for clinical outcomes. Patients with moderate lesions and FFR values ≥0.75 were randomly assigned to either PCI or deferred PCI with pharmacological treatment. After two years, event-free survival rates were comparable (deferred group: 89%, surgical group: 83%, *p* = 0.27). The incidence of myocardial infarction was significantly lower in the deferred group (2.2% vs. 10%, *p* = 0.03), and the revascularization rate did not significantly increase after 15 years of follow-up, indicating the potential for the FFR to guide PCI therapy.

For patients with CAD presenting more than two lesions, the FFR is essential for pre-PCI assessment. The Fractional Flow Reserve and Angiographic Multivessel Evaluation (FAME3) trial enrolled 1500 patients with CAD and triple-vessel coronary artery lesions [5]. Participants were randomized in a 1:1 ratio to either the PCI group, where FFR-guided drug-eluting stenting with zotarolimus was performed, or the coronary artery bypass grafting (CABG) group. The study demonstrated that FFR guidance can enhance PCI therapy by reducing the risk of unnecessary PCI procedures and improving clinical outcomes.

Despite the evidence supporting the ability of the FFR to increase diagnostic precision for myocardial ischemia and its value in diagnosing ambiguous cases and guiding PCI treatment [6], its use remains limited by its invasive nature and high cost. Recently, a noninvasive technique was explored that uses coronary computed tomographic angiography to simulate vascular flow, producing hemodynamic data equivalent to the FFR.

### 1.1 Fractional Flow Reserve by CT

First introduced by Heartflow in the US, computed tomography-derived fractional flow reserve (CT-FFR) is a noninvasive method of measuring the FFR based on coronary CT angiography (CCTA). The initial CT-FFR technique relied on computational fluid dynamics (CFDs), utilizing static CT image data to simulate cardiac blood pressure and velocity. FFR values were calculated using the Navier–Stokes equations [7]. However, the original full-sequence CT-FFR algorithm was complex and required data to be transferred to an off-site supercomputer for processing, significantly limiting its clinical application. This limitation persisted until the development of uCT-FFR analysis software, which reduced computational time. Subsequently, machine learning-based CT-FFR was developed [8]. This approach establishes the relationship between anatomical features and FFR values through deep learning of anatomical case models of coronary arteries with various stenotic branches. This advancement enables on-site application, reducing operational complexity and computation time. Deep learning-based CT-FFR can compute and analyze FFR values in real-time, leveraging the trained model directly [9].

### 1.2 Why We Need CT-FFR

With an increasing number of people needing elective cardiac catheterization procedures annually, a reliable cardiac catheterization gatekeeper could be extremely helpful for the patient population. Computed tomography (CT) is a widely utilized imaging technique for evaluating the coronary artery status in CHD to assess the anatomical narrowing of the coronary vessels properly. Nevertheless, the degree of coronary function of a lesion is not directly determined by its anatomic stenosis, and even CCTA with contrast agents cannot reveal further information about the degree of coronary function. Moreover, the use of contrast agents carries the risk of allergic reactions and potential renal impairment, particularly in patients with pre-existing kidney dysfunction [10]. Furthermore, the interpretation of CT scans can be significantly confounded by coronary calcifications and stents, impairing the diagnosis specificity and accuracy [11, 12]. Consequently, patients who have undergone CT may still require coronary angiography or invasive FFR assessments, which raises the patient’s radiation exposure. Actually, we can use the information from CT scans more effectively, thereby sparing patients from radiation and damage.

The CT-FFR value has been proven by numerous studies that have compared the sensitivity and specificity of CT-FFR against other procedures (Table 1, Ref. [13, 14, 15, 16, 17, 18]). In the Coronary Artery Stenosis and Prognosis Detection trial [13], at the individual patient level, CT-FFR achieved an accuracy, sensitivity, specificity, positive predictive value (PPV), and negative predictive value (NPV) of 90.4%, 93.6%, 88.1%, 85.3%, and 94.9%, respectively. At the vascular level, these results were 90.4%, 93.6%, 88.1%, 85.3%, and 94.9%, respectively. In both cases, the CT-FFR values were greater than the CCTA. The Blood Flow Reserve Fraction Diagnosis of Coronary Artery Disease trial [19], which included 11,202 patients, found that the extended basic model with CCTA and Agatston calcium score (CCS) had an area under the curve (AUC) of 0.876 (95% CI 0.829–0.923, *p* < 0.001), while the addition of CT-FFR increased the AUC to 0.929 (95% CI 0.895–0.962, *p* = 0.398). In the multicenter PLATFORM study [20], 584 CAD patients exhibiting symptoms were randomized to the computed tomography coronary angiography (CTCA) ± FFRct intervention or the ICA program testing technique. Among those who underwent pre-ICA, CTCA ± FFRct led to delayed ICA in 61% of cases. Significantly fewer patients had non-occlusive disease with ICA compared to pre-ICA (12% vs. 73%, *p* = 0.0001), suggesting that CT-FFR may reduce referrals for ICA and avoid unnecessary radiation exposure.

**Table 1.** **Diagnostic performance of the single CT-FFR compared with a multi-technology combination**.

Modality	n	Sensitivity	Specificity	PPV	NPV	AUC
	Pt	Pt_1_%	Pt_1_%	Pt_1_%	Pt_1_%	P
CCTA [13]	73	61.3	52.4	48.7	64.7	0.599
CT-FFR [13]	73	93.6	88.1	85.3	94.9	0.957
Severe calcification + CT-FFR [17]	38	66.7	75.0	70.6	71.4	0.740
CCTA + CT-FFR [17]	48	84.0	93.0	89.0	90.0	0.930
Fusion-FFR [14]	148	84.6	84.3	80.9	87.5	—
FAI + CT-FFR [15]	38	100.0	80.0	81.8	100.0	0.919
PCAT + CT-FFR [16]	146	65.0	87.0	76.0	81.0	0.875
CT-(Pd/Pa)rest [18]	20	85.0	91.0	92.0	83.0	0.870

AUC, area under the receiver operator characteristic curve; PPV, positive predictive value; NPV, negative predictive value; Pt, per-patient; CT-FFR, computed tomography-derived fractional flow reserve; CCTA, coronary CT angiography; CT, computed tomography; FAI, fat attenuation index; Pd/Pa, the resting distal-to-aortic coronary pressure ratio; PCAT, pericoronary adipose tissue.

For diagnostic performance, uCT-FFR showed an AUC per vessel of 0.94 (95% CI 0.90–0.97) in a study based on a computational fluid dynamics-based method comprising 299 moderately stenotic arteries in 246 patients with vascular ischemia-specific CAD [21]. CT-FFR is a useful diagnostic in “gray zone” lesions and performs well in identifying lesion-specific ischemia. In the study of noninvasive CT-FFR [22], two patients with an FFR value of 0.80 were chosen to determine the model parameters, and the remaining 18 patients were allocated to the validation group. CT-FFR had comparable diagnostic accuracy to FFR and a strong correlation, demonstrating the utility of incorporating noninvasive CT-FFR into clinical diagnosis.

A multicenter study investigated coronary-specific ischemia lesions in 324 arteries in 316 individuals [23]. At the vascular level, CT-FFR demonstrated a diagnostic sensitivity, specificity, and accuracy of 95.3%, 89.8%, and 92.0%, respectively. In contrast, CCTA exhibited a sensitivity, specificity, and accuracy of 96.4%, 26.4%, and 53.1%, respectively. CT-FFR outperformed CCTA in detecting ischemia overall (AUC: 0.95 vs. 0.74, *p* < 0.001), in intermediate lesions (AUC: 0.96 vs. 0.62, *p* < 0.001), and in “gray zone” lesions (AUC: 0.88 vs. 0.61, *p* < 0.001).

CT-FFR has correlated well with the PCI gold standard FFR in randomized trials with short observation periods, achieving comparable noninvasive diagnostic efficacy to the FFR [22, 23, 24]. Thus, support for CT-FFR is growing due to its improved algorithms and ability to be combined with other noninvasive tests to enhance diagnostic accuracy. CCTA, another noninvasive test, excels in anatomical diagnostics for identifying obstructive disorders and has advantages over myocardial perfusion imaging (MPI) and the more costly magnetic resonance imaging (MRI) and positron emission tomography (PET). Previous research has indicated that the degree of coronary stenosis does not always correspond to the degree of coronary ischemia. While CCTA visualizes stenosis severity, it does not provide information on physiological function. The introduction of CT-FFR significantly augments anatomical assessments, and combining CT-FFR with CCTA enhances diagnostic performance and prognostication. In a study using several models for the combined diagnosis of coronary artery disease, 202 patients with coronary artery disease received exercise electrocardiography, single photon emission computed tomography (SPECT/MPI), CCTA, CT-FFR, ICA, and FFR [25]. Multivariate logistic regression models were created to examine the impact of detection. The AUC for the base model (likelihood of CAD + exercise stress electrocardiography (ECG)) was 0.790 (95% CI 0.726–0.853, *p* < 0.001). Further, combining CCTA and CCS boosted the AUC to 0.876 (95% CI 0.829–0.923, *p* < 0.001), while adding CT-FFR increased the AUC to 0.929 (95% CI 0.895–0.962, *p* = 0.398). CT-FFR has been tested in several hospitals, although whether it can overcome the challenges of FFR and aid other clinical tests to improve the diagnostic efficacy of CAD remains to be seen.

Future advancements in noninvasive procedures will present more challenges for CT-FFR. For instance, the Ge team from Fudan University is currently comparing the guidance role of quantitative flow ratio (QFR) and CT-FFR in treatment plans. The Cecco team at Emory University is conducting research comparing the diagnostic performance of CT-FFR, CT-MPI, and PET-MPI. However, the precise and efficient CT-FFR remains dominant.

## 2. Limitations in CT-FFR

With technological advancements, the CT-FFR approach has been proven reliable in diagnosing coronary artery disease. It appears to have better specificity and PPV than both ICA and CTA [26]. Nevertheless, several issues with CT-FFR still need to be resolved before it can be observed as the gold standard. Numerous studies have addressed these challenges. Early in CT-FFR development, sophisticated CFD algorithms required analysis by supercomputers, resulting in long diagnostic times and the need for data transfer across countries, significantly limiting clinical usage. The CFD-based downscaled CT-FFR employs simplified Navier–Stokes equations to calculate pressure ratios, reducing calculation time to 30 minutes. The advent of machine learning-based CT-FFR and on-site workstations greatly expanded clinical application [14, 21, 27].

Many datasets are unsuitable for CT-FFR evaluation in clinical research, as CT-FFR requires high-quality image data [28]. Degradation in image quality can be caused by motion artifacts, low resolution, shear structures, valve opening and closing, and image noise [29, 30]. Motion artifacts are the primary cause of poor image quality and reduce as the heart rate becomes more stable, slower, or has more z-axis coverage [31]. Thus, to maximize the utility of the gathered data and improve diagnostic accuracy, patients should be evaluated before CT-FFR.

Prior research has found no significant distinction in CT-FFR diagnostic proficiency between severe and mild calcification [32, 33, 34]. However, it was observed that in cases of severe coronary calcification (Agatston score ≥400), calcified plaque may cause the coronary lumen to become obscured, limiting CFD analysis. The likelihood of this occurring increases with the Agatston score, decreasing diagnostic performance [28]. Extensive manual correction is needed for highly calcified images, and skilled analysts must adjust to ensure accuracy [35, 36, 37]. Furthermore, diffuse coronary calcification has a major impact on CT-FFR imaging, and the architecture of the calcium ring impairs the visibility of the coronary lumen. Hence, diagnostic performance is considerably compromised when a lesion is more than semi-circularly calcified [38, 39].

The CAD Reporting and Data System (CAD-RADS) standardizes CCTA reports and stratifies patients based on the degree of coronary stenosis. Research has indicated that CAD-RADS is a valuable prognostic tool for major adverse cardiovascular events (MACEs) [40, 41], and predictive CAD performs better as a diagnostic factor than independent factors such as coronary artery calcium score (CACS) and high-risk plaques [42]. The implementation of CAD-RADS was shown to guide treatment from coronary CTA to subsequent care in patients presenting with chest discomfort and mixed degrees of stenosis [43, 44]. To enhance the accuracy of CAD diagnosis, some researchers suggest combining CT-FFR with CAD-RADS. A study of 1145 patients undergoing CTCA revealed that CT-FFR with CAD-RADS had comparable sensitivity (92.7% vs. 82.9%, *p* = 0.102) but poorer specificity (75.5% vs. 92.7%, *p* < 0.001) and accuracy (78.4% vs. 91%, *p* < 0.001) [45]. Therefore, rather than enhancing the CAD-RADS efficacy, CT-FFR reduces its diagnostic capabilities. Yang *et al*. [46] found that in patients with moderate coronary stenosis (CAD-RADS 3), CT-FFR increased sensitivity but lowered specificity and accuracy. Similarly, in CAD-RADS 2–4, CT-FFR decreased accuracy and specificity. The specificity of CT-FFR declined in cases of significant coronary stenosis (CAD-RADS 4) [45, 46].

## 3. Defect Improvement

Numerous studies have demonstrated that CT-FFR’s advantages, including noninvasiveness, ease of use, low radiation dose, and superior diagnostic performance, make it a strong contender for the “gatekeeper” role in the cardiac catheterization laboratory [10, 13, 19]. However, practical experience has also revealed certain limitations associated with CT-FFR. If these issues can be addressed, the future clinical application of CT-FFR appears promising (Table 1).

### 3.1 Technique Improvement

Selecting an algorithmic model is crucial in using CT-FFR to compute the FFR values using algorithms rather than guidewires. The original CFD-based CT-FFR algorithms were complicated and computationally laborious. In the trials including Navigating the X-Factor in Diabetes (NXT), Prospective Multicenter Imaging Study for Evaluation of Chest Pain (PROMISE), and Action in Diabetes and Vascular Disease: Preterax and Diamicron Modified Release Controlled Evaluation (ADVANCE), between 3% and 33% of the CFD-based CT-FFR pictures were discarded due to low quality [47, 48, 49]. However, machine learning-based CT-FFR incorporates various image reconstruction algorithms that do not compromise lumen and plaque identification. The CT-FFR values do not differ across varying calcification levels and are unlikely to be impacted by calcified plaque artifacts [50]. In a total of 344 calcified lesions, a study found that machine learning (ML)-based CT-FFR had an overall sensitivity of 0.84, specificity of 0.94, and accuracy of 0.90. It also found that ML-based CT-FFR performed better as a diagnostic tool than CTA for mild, moderate, and severe stenoses [51]. As the use of machine learning in CT-FFR develops, more focus is on applying sophisticated deep learning techniques, such as convolutional neural network (CNN) [52]. Multiple studies have found favorable outcomes and low levels of heterogeneity in the comprehensive diagnostic performance of CT-FFR based on deep learning [27, 53, 54]. According to some research, variations in microcirculatory resistance significantly impact CT-FFR computations [55, 56]. If calculations of individual microcirculatory resistance changes are incorporated, the diagnostic accuracy of CT-FFR will be increased. Hence, to get an individual distribution of coronary resting blood flow, Zhang *et al*. [57] proposed using the anisotropic growth law of the 7/3 index to compute the CT-FFR. This method evaluated 121 arteries and produced an accuracy of 91.74%.

Calculations previously limited to supercomputers can now be completed on-site, and technological improvements have coincided with shorter CT-FFR examination durations. In 2017, to further expand its profit margins, Heartflow partnered with Siemens, GE, and Philips to integrate their CT-FFR technology into the CT scanners of the aforementioned companies. These businesses’ products performed well in diagnostics. Siemens (contrast-FFR (cFFR) version 1.4) showed a sensitivity of 82%–86% and a specificity of 63%–83% [58, 59]. Toshiba (Toshiba Medical Systems, Tokyo, Japan) offers CT-FFR with a sensitivity of 83% and a specificity of 84%–88% [54, 60]. A study by Fujimoto *et al*. [61] using on-site CT-FFR provided a high sensitivity of 91% and a moderate specificity of 78%.

After starting in the United States, the technology has now spread to the European Union, Japan, and the United Kingdom. The next year, as these nations gradually placed the product in the purview of health insurance coverage, CT-FFR devices outfitted with Heartflow software established a monopoly in the United States, the United Kingdom, and Japan [62]. However, apart from the aforementioned countries, the technology remains generally inaccessible. However, as AI has grown, several Chinese businesses and groups have formed to work on CT-FFR technology development. They have launched products with detection efficacy comparable to Heartflow, faster processing times, and more adaptable application situations by relying on multi-layer deep learning and artificial intelligence algorithms (Table 2, Ref. [13, 21, 27, 47, 53, 58, 63, 64]). While Heartflow remains the primary service mode of the CT-FFR in the United States, Siemens and China’s Zhang Longjiang team have started testing a more convenient and straightforward on-site CT-FFR, enabling fast access to results. Guo *et al*. [27] launched a completely automated CT-FFR and significantly streamlined the operating procedure based on on-site CT-FFR. Compared to the existing manual CT-FFR technology in hospitals, it has shortened the computation time while still maintaining the accuracy of the calculations (0.82). On-site CT-FFR research is conducted mainly in Germany, Japan, and China.

**Table 2.** **Comparison of different CT-FFR suppliers and products**.

Company/Organization	Production	Characteristic	Individuals		Vessels		Approval
			Sensitivity (%)	Specificity (%)	Sensitivity (%)	Specificity (%)	
HeartFlow [47]	FFRCT	Primary 3D-CFD	86	79	84	86	FDA (2014)
Siemens [58]	cFFR	On-site	87	86	—	—	Cooperate with HeartFlow (2017)
Toshiba [53]	CT-FFR	Reduced 3D-CFD	77.8	76.8	—	—	—
RuiXin [63]	RuiXin-FFR	Multi-layer deep learning	87	88	—	—	MDR (2023), NMPA (2021)
United Imaging [21]	uCT-FFR	AI computing	89	91	—	—	NMPA (2024)
Shukun Technology [64]	Shukun-FFR	AI computing	96.2	93.1	83.6	96.3	NMPA (2023)
YueYing	MaiYing®esFFR	3D-CFD	—	—	—	—	NMPA (2022)
KeYa [13]	CT-FFR	3D-CFD	93.6	88.2	93.9	90.4	FDA (2022), NMPA (2020), CE (2018),
Affiliated Jinling Hospital, Medical School of Nanjing University [27]	CT-FFR	Fully automatic, on-site	84	81	—	—	Under testing (2024)

FDA, Food and Drug Administration (United States); MDR, Medical Device Regulation (European Union); NMPA, National Medical Products Administration; CE, Conformité Européenne; CFD, computational fluid dynamics; CT-FFR, computed tomography-derived fractional flow reserve; FFR, fractional flow reserve; 3D, three dimensional; uCT-FFR, ultra-high resolution computed tomography-fractional flow reserve; FFRCT, fractional flow reserve derived from standard acquired coronary computed tomography angiography datasets; cFFR, contrast-FFR; AI, artificial intelligence.

In outpatient and emergency settings, the worth of CT-FFR technology is further recognized. Recently, at Massachusetts General Hospital, Harvard Medical School, Randhawa *et al*. [65] evaluated the effects of CT-FFR on coronary stenosis evaluation and the usage of ICA and PCI in inpatient, outpatient, and emergency patients over 3 months. In the inpatient and emergency department cohorts, 331 out of 1218 patients (27.2%) had significant stenosis, while in the outpatient cohort, 406 out of 1767 patients (23.0%) were identified with notable stenosis. Among these patients identified with stenosis by CCTA, those who underwent CT-FFR showed a significantly reduced rate of ICA and PCI compared to those who did not undergo CT-FFR. By eliminating the need for extra testing, on-site CT-FFR reduces contrast use and improves diagnostic accuracy for patients with stable CAD who are being considered for conversion to ICA [66].

### 3.2 Multi-technology Combination

The low resolution of coronary CTA pictures limits the use of CT-FFR. In contrast, optical coherence tomography (OCT) offers high-resolution images and, thus, more accurate coronary artery and atherosclerotic plaque information [67]. Fusion-FFR, the CFD-based coronary CTA-OCT fusion image calculation of FFR, has demonstrated a stronger correlation with FFR than CT-FFR with FFR. Additionally, the area under the working characteristic curve in subjects assessing functionally significant stenosis was higher than that of CT-FFR (0.90 vs. 0.83, *p* = 0.024) and had better accuracy, specificity, and PPV than CT-FFR [14]. Results measured by CT-FFR in diastole (D) differ from those measured in systole (S). In most false-negative lesions measured in S, the lumen is relatively narrow, and slight artifacts are typically present in smaller target lesions, increasing the risk that the lesion will be lost during coronary artery extraction. However, calculations can be performed using D data to reduce the adverse effects of artifacts on image quality [68]. The measuring position also has an impact on CT-FFR results. FFRCT (fractional flow reserve derived from standard acquired coronary computed tomography angiography datasets)-1 cm (0.91) and FFRCT-2 cm (0.91) have higher AUCs for distinguishing lesion-specific ischemia than FFRCT-3 cm (0.89) and FFRCT-4 cm (0.88) (both *p* < 0.05). Of these, only FFRCT-2 cm was an independent predictor of MACEs (hazard ratio: 0.957 (95% CI 0.925~0.989); *p* = 0.010) [69]. Some research suggests that a flow-based approach to coronary CTFFR (CTQFFR) resolves the CT-FFR correlation downstream of coronary artery stenosis with the chosen location, potentially increasing the reliability of CT-FFR near the adventitia [70]. The decline in proximal-to-distal vessels, influenced by vessel bifurcation and vascular flow parameters [71, 72], is another frequently observed imaging feature. At the distal end of the no-apparent CAD vessels, there is a drop in CT-FFR, affecting the assessment of ischemia at the distal end. The V/L ratio was defined as the lumen volume divided by the vessel length. One potential solution to address this is to introduce a metric called V/L ratio assessment, which compares the V/L of the target vessel to that of a matching healthy vessel [71].

As demonstrated by prior research, the CT-FFR picture quality is negatively impacted by diffuse and severe calcification [15, 29, 73]. Researchers are exploring combining CT-FFR with other methods to address this issue. Considered a vessel wall thermometer, perivascular fat attenuation index (FAI) in patients with acute coronary syndrome (ACS) varies with plaque advancement and rises with vessel ischemia [74, 75, 76]. Patients with diffuse calcification related to a vascular inflammatory response are more likely to have high perivascular FAI, which can assist in detecting events in patients with severe calcification [15, 75, 77]. Compared to conventional CCTA, the combined evaluation of FAI and CT-FFR enhances the identification of culprit lesions in subsequent ACS events [78]. CT-FFR can overcome the diagnostic interference caused by calcified plaques and work in conjunction with baseline quantitative plaque analysis to identify plaque progression (PP) [79] and with the CCTA diagnostic and treatment system to optimize non-obstructive CAD management. These combinations are advantageous for prevention and early diagnosis. Absolute myocardial blood flow (MBF) quantification can be determined by dynamic CT myocardial perfusion imaging (CT-MPI). Further, not only does dynamic CT-MPI assess microvascular dysfunction without being impacted by calcification, but it also outperformed CT-FFR and high-risk plaque (HRP) features as the best predictor of MACEs [80].

Another significant factor affecting the FFR measures is endothelial dysfunction caused by vascular inflammation. When arteries are at maximum congestion, diastolic function is compromised, resulting in reduced relative pressure and false-positive FFR values. However, the recently proposed imaging parameter pericoronary adipose tissue (PCAT) [74] can compensate for this limitation, which reflects active perivascular inflammation. Studies have demonstrated that incorporating PCAT radiomics modeling into CT-FFR may enhance its ability to distinguish flow-restricted lesions from non-flow-restricted lesions [16, 74]. However, the absolute attenuation of PCAT can be influenced by various factors, meaning it is unlikely to reflect the relationship between ischemic stenosis and PCAT simply. As a result, PCAT should be used in conjunction with CT-FFR to achieve a more accurate assessment [16].

CT-FFR has yet to be extensively studied for evaluating myocardial ischemia status. Combining CT-FFR and CCTA has been proposed to concurrently assess myocardial blood supply at both functional and anatomic stenosis levels, providing a comprehensive diagnosis. When identifying patients with flow-restricted CAD, as defined by ICA + stress single photon emission computed tomography (SPECT/MPI), the combination of these two modalities was similar to CT perfusion scanning plus CCTA [81, 82]. The sensitivity and specificity for identifying myocardial ischemia were 81% and 96%, respectively, with good diagnostic efficacy (AUC = 0.92, *p* = 0.01). This combination was also more accurate and economical because it minimizes injuries and avoids the unnecessary use of contrast agents [17]. The CT-FFR plus CCTA combination further enhanced the diagnostic capacity for acute myocardial infarction (AMI), improving the AUC to 0.914 when compared to the CT-FFR model alone or the CCTA model alone [83].

Despite the lack of research into “gray regions”, studies have shown that the diagnostic efficacy of CT-FFR in individuals with FFR values in the 0.75–0.80 range declines [58, 84, 85]. Hence, increasing the percentage of subsequent hemodynamic reconstruction may be likely when using the change in fractional flow reserve derived from CT across coronary stenoses (ΔFFRCT) to maximize the discrimination of hemodynamic situations in this population [86]. The resting distal-to-aortic coronary pressure ratio (Pd/Pa) is considered a simpler measure than FFR for determining the severity of stenosis. The resting distal-to-aortic coronary pressure ratio (Pd/Pa) can be as accurate as 80% of the criteria for iFR versus FFR [87]. With a FFR value of <0.80 used as a reference, Li *et al*. [88] proposed a noninvasive resting Pd/Pa calculation method (CT-(Pd/Pa)rest) based on a noninvasive coronary CTA. The diagnostic performance of CT-(Pd/Pa)rest was satisfactory, with a sensitivity of 0.85, a specificity of 0.91, a PPV of 0.92, and a NPV of 0.83. When the test results are in the “gray zone”, the CT-FFR may be less reliable in guiding PCI and could be replaced by CT-(Pd/Pa) rest [88].

A combination of technologies can improve the identification of the eventual development of obstructive CAD in patients with early moderate illness. With normal cardiac biomarkers and electrocardiograms showing no signs of ischemia, 10% of patients first diagnosed with acute myocardial infarction had chest pain and were considered to be at low-to-moderate risk [18]. At this stage, FAI and CT-FFR can be used for early identification. Combining CT-FFR and CACS can help detect early coronary stenosis and predict mild stenosis by vector machine modeling [89]. Notably, research indicates that the degree of coronary stenosis and risk of obstructive CAD increase with the CACS.

## 4. CT-FFR in other Coronary-related Diseases

Aortic stenosis (AS) is a prevalent heart valve condition that can result in reduced leaflet opening and stenosis that indirectly affects cardiac function. Transcatheter aortic valve replacement (TAVR) is a common procedure for treating AS [90, 91]. According to Aquino *et al*. [92], there is no correlation between CT-FFR and the incidence of adverse cardiac events in TAVR. However, univariate analyses revealed that other common predictive risk factors were unrelated to all-cause mortality. This could be because patients with TAVR frequently have multiple comorbidities that result in non-cardiac deaths. In contrast, CT-FFR was able to predict cardiac death; patients with a CT-FFR of 0.75 or below before TAVR had a 4-fold greater chance of MACEs [92]. With an 82.6% diagnosis accuracy and an AUC of 0.87, CT-FFR based on traditional TAVR-CT lowers the number of ICAs in patients with AS [93]. Nonetheless, prospective trials are required to validate the potential of CT-FFR assessment before regular TAVR in patients suffering from AS.

TAVR patients with compromised hepatic and renal metabolic function and/or liver and kidney transplants have a higher metabolic burden on their liver and kidneys due to the use of relatively high radiation doses in CT-FFR [94]. It is anticipated that the high sensitivity and specificity of CT-FFR for CAD, in conjunction with machine learning algorithms and on-site CT-FFR, will replace ICA before liver transplantation [95], minimizing radiation dose damage and the effects of contrast media on the liver and kidneys.

Individuals with diabetes have twice the risk of developing MACEs compared to those without the disease [96]. For those with diabetes, screening for CAD is essential. Research has shown that CT-FFR is a highly reliable independent predictor of MACEs in patients with diabetes. The incorporation of CT-FFR into the initial diabetic low attenuation plaque (LAP) and hemoglobin A1c (HbA1c) prediction models enhanced the model’s identification of patients at an elevated risk of developing diabetic MACEs [84]. Global trans-lesional CT-FFR gradient (GΔCT-FFR) was used to predict clinical outcomes in individuals with non-obstructive CAD and diabetes mellitus at a 5-year follow-up. After a median follow-up of 57.3 months of 1215 patients (60.1 ± 10.3 years, 53.7% male), 11.3% had developed MACEs, and GΔCT-FFR proved its prognostic value (HR: 2.88, 95% CI 1.76~4.70, *p* < 0.001) [97]. For diabetes patients, all of these variables help determine early intervention therapies that may reduce unfavorable outcomes.

Patients with myocardial bridging (MB), a frequent congenital coronary artery variation, are more likely to have proximal artery atherosclerotic plaque aggregation. Zhou *et al*. [98] conducted a retrospective study to investigate the use of CT-FFR in MB and found that the two best predictors of proximal plaque formation in left anterior descending (LAD) MB were DCT-FFR and CT-FFR values. A better predictor of proximal plaque formation in patients with LAD MB would be combining MB morphological features with CT-FFR features. Tang *et al*. [99] completed a retrospective study on 94 samples from patients with a right coronary artery originating from the left coronary sinus (R-ACAOS) and found that the starting level, intramural alignment, and fissure-like orifice could all predict hemodynamic abnormalities with accuracy of 0.69, 0.71, and 0.81, respectively. These findings suggest that CT-FFR could be a useful tool for assessing the functional characteristics of patients with R-ACAOS.

The great potential of CT-FFR will make it possible to diagnose many more disorders in addition to those listed above. While previous research has concentrated on acute coronary syndromes, the Kofoed and Wang teams are now filling in the gaps in the utilization of CT-FFR for this illness by conducting clinical studies on it to help guide treatment methods for chronic coronary syndromes (Kofoed, 2021. In Study Details | CT Stress Myocardial Perfusion, Fractional Flow Reserve and Angiography in Patients With Stable Chest Pain Syndromes | ClinicalTrials.gov). In the meantime, Qiang Xue’s group is examining the effects of CT-FFR on follow-up interventions for patients with post-stent coronary restenosis by comparing standard examinations with the CT-FFR test (Qiang Xue, 2022. In Study Details | CT-FFR-guided Strategy for In-stent Restenosis | ClinicalTrials.gov).

## 5. Conclusions

The advent of invasive FFR has significantly impacted the field of interventional imaging. The diagnostic criterion of infiltrative FFR <0.80 has been widely utilized in clinical diagnosis and treatment and in the investigation of alternative imaging techniques. Due to its exceptional sensitivity and specificity, the FFR has gained global recognition as the gatekeeper of interventions. The recently developed CT-FFR has shown superior diagnostic performance compared to widely used MPI and echocardiography, and it appears to have greater sensitivity and specificity than coronary CTA alone. However, CT-FFR, despite its noninvasive nature, remains expensive and demanding in terms of image quality, posing a challenge to its adoption as the new gold standard.

Several challenges for CT-FFR must be addressed. Firstly, merely altering the CT-FFR technique will not overcome the high-quality requirements for image acquisition, nor will it adequately assess conditions such as severely calcified coronary arteries, myocardial ischemic disease, or plaque characteristics. These issues necessitate integration with other diagnostic factors. Secondly, contrast agents are frequently used for diagnosing patients with liver and kidney transplants, myocardial ischemia, obstructive or non-obstructive CAD, and AS. Therefore, more prospective research is required to evaluate whether CT-FFR can fully replace ICA in these scenarios. Thirdly, CT-FFR has yet to be widely adopted, and many hospitals need more equipment for optimal image capture. Further application and investigation of CT-FFR technology, particularly those based on machine learning and deep learning, are required.

**Fig. 1.**
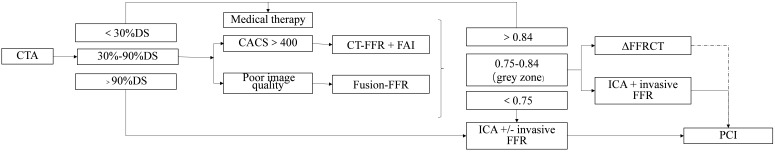
**A futuristic flowchart founded on the continued approval and validation of CT-FFR as the gold standard of care**. After CT angiography (CTA), patients will be triaged to medical therapy after <30% diameter stenosis (DS), directly to the catheterization laboratory with >90% DS, and to fractional flow reserve from computed tomography (CT-FFR) with findings in the 30% to 90% range. Further triage will follow CT-FFR. Thereafter, it will be combined with the fat attenuation index (FAI) examination. If there is extensive calcification in the vessels and the picture quality is poor, it will be further combined with Fusion-FFR to enhance it. Patients in the “gray zone” of >0.84 will be referred for medical treatment. Those in the “gray zone” of 0.75 to 0.84 may be diagnosed using invasive FFR assessment and invasive angiography after receiving subpar results from FFRCT. Patients with a CT-FFR under 0.75 will have angiography; invasive FFR will only be carried out in diffuse disease cases requiring further ischemia localization. In patients with >90% DS, invasive FFR will be used if the angiographic results show less severe abnormalities than the CTA. Lastly, patients with an invasive FFR of ≤0.80 will be recommended for percutaneous coronary intervention (PCI) and medicinal therapy if FFR >0.80. CACS, coronary artery calcium score; FFR, fractional flow reserve; ΔFFRCT, change in fractional flow reserve derived from CT across coronary stenoses; ICA, invasive coronary angiography.

Effective CT-FFR has the potential to reduce unnecessary ICA procedures, enhance diagnostic efficacy, and lower the risk of injuries related to invasive testing. Although not yet specifically recommended by clinical guidelines, CT-FFR techniques are gradually being introduced into clinical practice as ongoing trials demonstrate their utility. A futuristic flowchart based on further validation and acceptance of CT-FFR as the standard of care is presented in Fig. 1.

The precision and efficiency of CT-FFR operations have improved from the first generation of supercomputers computing hydrodynamics to the combination of multidimensional, deep learning, and artificial intelligence. There have been numerous improvements to CT-FFR. Following on-site CT-FFR trials conducted by Japan, Germany, Hungary, Holland, China, South Korea, and other countries, the Longjiang Zhang team in China initially introduced a fully automated CT-FFR technology based on on-site CT-FFR. This technology has a high calculation success rate and reduces operation time to one-third of manual operation. Furthermore, multimodal imaging fusion can be achieved, and a more thorough vascular assessment will be obtained by combining CT-FFR with other imaging modalities, including CCTA and intravascular ultrasound (IVUS). While CT-FFR has gained broader adoption in the United States, Germany, Japan, and elsewhere, there remains significant room for growth in numerous countries, including China. As the technology matures and clinical evidence accumulates, CT-FFR, which is noninvasive and reduces the medical costs associated with FFR, will gradually be accepted by many more countries and regions. CT-FFR, as the gold standard for diagnosis, will also become applicable to a wider range of areas.

## Data Availability

The datasets generated and analyzed during the current study are available in the Pubmed and Clinicaltrials.gov.

## Abbreviations

CT-FFR, computed tomography-derived fractional flow reserve; FFR, fractional flow reserve; MACE, major adverse cardiovascular event; TAVR, transcatheter aortic valve replacement; CAD, obstructive coronary artery disease; CHD, coronary heart disease; CTA, CT angiography; PCI, percutaneous coronary intervention; FAME3, Fractional Flow Reserve and Angiographic Multivessel Evaluation trial; CCTA, coronary CT angiography; CFD, computational fluid dynamics; uCT-FFR, ultra-high resolution computed tomography-fractional flow reserve; PPV, positive predictive value; NPV, negative predictive value; AUC, area under the curve; CTCA, computed tomography coronary angiography; CAD-RADS, CAD Reporting and Data System; CACS, coronary artery calcium score; CTCA, computed tomography coronary angiography; NXT, Navigating the X-Factor in Diabetes; PROMISE, Prospective Multicenter Imaging Study for Evaluation of Chest Pain; ADVANCE, Action in Diabetes and Vascular Disease: Preterax and Diamicron Modified Release Controlled Evaluation; CTQFFR, flow-based approach to coronary CTFFR; FAI, fat attenuation index; MBF, myocardial blood flow; CT-MPI, CT myocardial perfusion imaging; ΔFFRCT, change in fractional flow reserve derived from CT across coronary stenoses; Pd/Pa, the resting distal-to-aortic coronary pressure ratio; GΔCT-FFR, global trans-lesional CT-FFR gradient; LAP, low attenuation plaque; MB, myocardial bridging; R-ACAOS, right coronary artery originating from the left coronary sinus.
